# Synthesis and Formulation of PCL-Based Urethane Acrylates for DLP 3D Printers

**DOI:** 10.3390/polym12071500

**Published:** 2020-07-05

**Authors:** Hsuan Chen, Shyh-Yuan Lee, Yuan-Min Lin

**Affiliations:** 1 Department of Dentistry, National Yang-Ming University, No 155, Section 2, LiNong Street, Beitou District, Taipei 112, Taiwan; tess1994131@gmail.com (H.C.); sylee@ym.edu.tw (S.-Y.L.); 2 Department of Stomatology, Veterans General Hospital Taipei, No. 201, Section 2, Shipai Road, Beitou District, Taipei 112, Taiwan; 3 Department of Dentistry, Taipei City Hospital, No.145, Zhengzhou Rd., Datong District, Taipei 103, Taiwan

**Keywords:** polyurethane acrylate, 3D printing, digital light processing, photopolymerization, biodegradable resin, PCL-based polyurethane

## Abstract

In this study, three PCL-based polyurethane acrylates were synthesized and further formulated into twelve resins for digital light processing (DLP) 3D printing. Three PCL diols with different molecular weights were synthesized via ring-opening reaction of ε-caprolactone on diethylene glycol, with the catalyst stannous octoate. Isophorone diisocyanate (IPDI) was reacted with 2-hydroxyethyl acrylate (2-HEA) and the PCL diols form PCL-based polyurethane acrylates. Twelve resins composed of different percentages of PCL-based polyurethane acrylates, poly (ethylene glycol) diacrylate (PEGDA), propylene glycol (PPG) and photo-initiator were further printed from a DLP 3D printer. The viscosities of twelve resins decreased by 10 times and became printable after adding 30% of PEGDA. The degree of conversion for the twelve resins can reach more than 80% after the post-curing process. By changing the amount of PEGDA and PPG, the mechanical properties of the twelve resins could be adjusted. PUA530-PEG-PPG (70:30:0), PUA800-PEG-PPG (70:30:0), and PUA1000-PEG-PPG (70:30:0) were successfully printed into customized tissue scaffolds. Twelve PCL-based polyurethane photo-curable resins with tunable mechanical properties, cytotoxicity, and degradability were successfully prepared. With the DLP 3D printing technique, a complex structure could be achieved. These resins have great potential for customized tissue engineering and other biomedical application.

## 1. Introduction

Polyurethanes (PU) have been widely applied in biomedical applications for decades. Because of the customizable mechanical properties, biocompatibility and biodegradability, polyurethanes have been used for numerous biomedical applications, such as artificial hearts, medical devices, blood bags, scaffolds and even dental aligners [1]. So far, photo-curable polyurethanes with adequate cytocompatibility and degradability for digital light processing (DLP) 3D printing technology were scarcely investigated and also rarely available on the market [2,3]. 

PU can be synthesized by the reaction of diisocyanates with diols. In this study, polycaprolactone (PCL) were chosen as the diol. PCL has also been widely used in the biomedical field, due to its excellent biocompatibility [4,5]. With the development of 3D printing technologies, researchers have started to synthesize PCL-based polyurethanes with 3D printing in recent years [6]. Many of the previous studies reported the thermoplastic polyurethane for fused deposition modeling (FDM) method rather than photo-curable polyurethanes for DLP 3D printers. 

For the formulation of 3D printing resins, poly (ethylene glycol) diacrylate (PEGDA) and propylene glycol (PPG) were added to PCL-based urethane acrylate to decrease the viscosities and tune the mechanical properties in this study. PEGDA is a low-toxic water soluble and flexible monomer, and has been adopted as scaffold materials using vat polymerization processes in many studies [7,8,9]. PPG is a colorless and nearly odorless viscous liquid. It has been used in a variety of applications such as thermoset composites, food chemistry, food processing equipment, cosmetics, pharmaceuticals, and even biodegradable PU [10]. Via pyruvic acid, PPG could be metabolized into carbon dioxide in human body. PPG was therefore approved by FDA for using in food products [11]. Apart from this, to improve flexibility of the composite, PPG was also used to improve the elasticity of chitosan-based films [12].

The aim of this study is to prepare a polyurethane-based photo-curable resin with flexibility, biocompatibility and degradability for DLP 3D printing technology. The polyurethane acrylates were synthesized with isophorone diisocyanate (IPDI), PCL diol and 2-HEA. PCL diols were synthesized in three different structures via ring-opening reaction of ε-caprolactone (ɛ-CL) on diethylene glycol (DEG), with the catalyst stannous octoate. In order to decrease the viscosities of the printable resin and tune different mechanical properties of final products, different ratios of PEGDA and PPG were adding to prepare twelve different resin formulations. 

## 2. Materials and Methods

### 2.1. Synthesis of PCL-Diols

**Figure 1:**
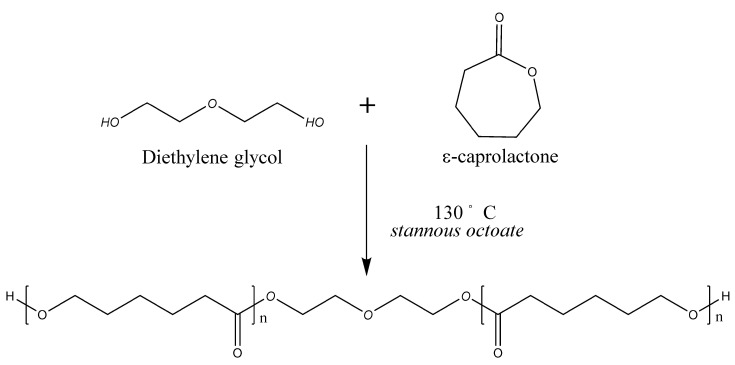
The synthesis process of polycaprolactone (PCL) diol. PCL diols were synthesized via ring-opening reaction of ε-caprolactone on diethylene glycol, with the catalyst stannous octoate (24 h, 130 °C).

Three different PCL diols were synthesized via ring-opening polymerization of ε-caprolactone (ε-CL) in the presence of diethylene glycol (DEG) (Figure 1). For the synthesis of PCL530-diol, ε-CL and DEG were reacted at 1:4 molar ratios of DEG to ε-CL. Tin(II) 2-ethylhexanoate at 0.1% *w*/*w* was added as the catalyst. The reaction was carried out at 130 °C for 24 h. For synthesis of PCL800-diol and PCL1000-diol, DEG was reacted with ε-CL at a 6.1:1 and 8.1:1 molar ratios, respectively. 

### 2.2. Synthesis of PCL-Based Urethane Acrylates 

**Figure 2:**
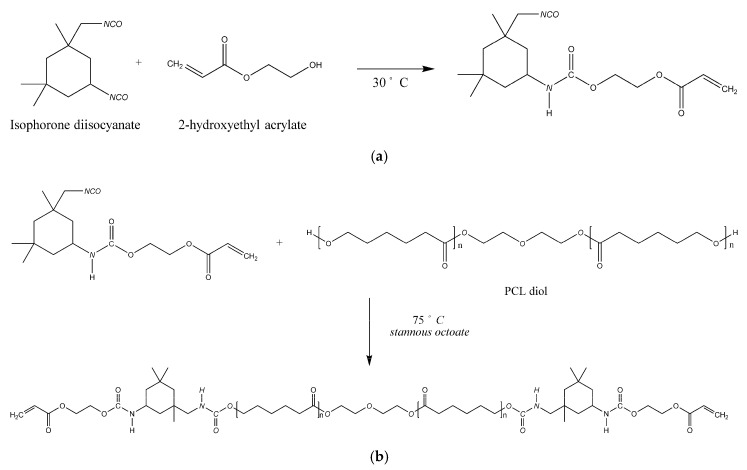
Synthesis processes of polyurethane acrylates. (**a**) In the first step, 2-HEA reacted with IPDI. (**b**) In the second step, the solution synthesized in the previous step reacted with PCL diol.

Three polyurethane acrylates (PUA) were synthesized from isophorone diisocyanate (IPDI), 2-hydroxyethyl acrylate (2-HEA) and three PCL diols at 2:2:1 molar ratio (Figure 2). 2-HEA was added dropwise to IPDI at 30 °C. The reaction kept running for 1.5 h. Then, the temperature was raised to 75 °C, and the PCL diol was added dropwise to the solution with the catalyst Tin(II) 2-ethylhexanoate and reacted for 12 h to form the polyurethane acrylates. The synthesized polyurethane acrylates that contain PCL530 diol, PCL 800 diol and PCL1000 diol were called m-PUA530, m-PUA800 and m-PUA1000, respectively. The prefix “m” stands for monomer. 

### 2.3. Formulation of Resins and 3D Printing 

12 resin formulations were prepared by mixing different ratios of m-PUA, polyethylene glycol (600) diacrylate (PEG600DA, M_n_ 600), polypropylene glycol (PPG, M_n_ 450) and 3% *w*/*w* diphenyl (2,4,6-trimethylbenzoyl) phosphine oxide (TPO) at 60 °C in the dark overnight, as shown in Table 1.

**Figure 3:**
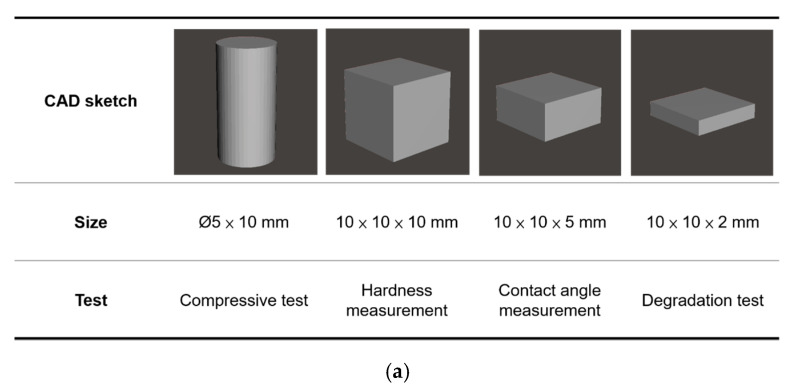

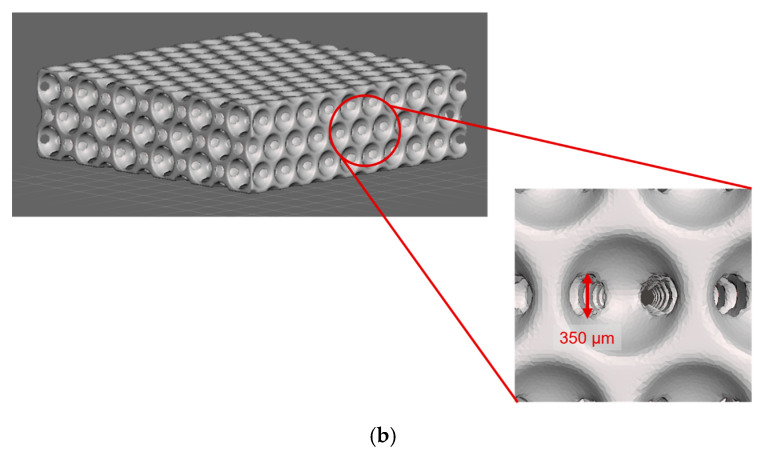
(**a**) The CAD sketch and the sizes of specimens for the compressive test, the hardness measurement, the contact angle measurement and the degradation test. (**b**) The design of the scaffold printed in this study.

The test specimens (Figure 3a) and scaffolds (Figure 3b) were designed in Autodesk Meshmixer (Autodesk Inc., San Rafael, CA, USA) software. 12 resins formulations were printed using a MiiCraft+ 3D printer (Young Optics, Hsinchu City, Taiwan). Printing parameters was set as the following: 50 μm of layer thickness, 4 s of curing time and 5 s of base layer curing time. After the specimens or the scaffolds was printed completely, a DC80H ultrasonic cleaner (Delta, Taipei City, Taiwan,) with 95% ethanol (Delight Ethanol Co., Taoyuan City, Taiwan) was used to remove the residual resin. Finally, the post-curing process was performed. The model was placed in a Form Cure post-curing box (Formlabs, Somerville, MA, USA), which was equipped with 39 W LED power and emitted 9.1 W LED radiant power, for 15 min at 60 °C. Pure PUAs were prepared for being the comparisons in the following characterizations. Because the viscosities of PUA are too high to be printed, the samples of PUA were prepared by a mold. The pure PUA with 3% TPO was poured into a polytetrafluoroethylene (PTFE) mold and the upper side and the down side were covered with microscope slides. Each side was cured for 30 s respectively by 405 nm near UV light.

### 2.4. Nuclear Magnetic Resonance (NMR) Analysis of PCL Diol and PUA

For characterizing the chemical structures of synthesized PCL diol and m-PUA, the ^1^H nuclear magnetic resonance (NMR) (400 MHz) spectra were recorded on an Avance III 400 NMR spectroscopy (Bruker, Billerica, MA, USA). Chemical shifts at 7.26 ppm stands for the solvent peaks of CDCl_3_. Topspin 3.0 software was used to delimit and integrate the peaks on the spectra.

### 2.5. Fourier-Transform Infrared (FTIR) Analysis PCL Diol and PUA

#### 2.5.1. Identification of Synthesized PCL Diol and m-PUA

In order to identify the functional groups of synthesized PCL diol and m-PUA, a Fourier-transform infrared (FTIR) spectrometer was used. FTIR analyses were carried out on a NICOLET iS5 FTIR spectrometer (Thermo Fisher Scientific, Waltham, MA, USA) equipped with zinc selenide (ZnSe) plate for iD3 attenuated total reflectance (ATR). 

#### 2.5.2. Calculation of Degree of Conversion of PUA and Resin Formulations 

The degree of conversion (DC) of three polymerized PUA and twelve differing printed formulas were obtained by using the FTIR. Each material was measured at three time points, including before polymerization, after printing and after post-curing process. The degree of conversion was calculated for each sample using the following equation:

$$\mathrm{DC}\;\left(\%\right)=\frac{\left(\frac{A_{monomer}}{C=O_{monomer}}\right)-\left(\frac{A_{polymer}}{C=O_{polymer}}\right)}{\frac{A_{monomer}}{C=O_{monomer}}}\times 100$$

where *A_monomoer_* is the absorbance at 810 cm^−1^ before exposure to the UV light, *A_polymer_* is the absorbance at 810 cm^−1^ after exposure to the UV light, *C=O_monomer_* and *C=O_polymer_* are the absorbances within 1720–1730 cm^−1^ of carbonyl stretching before and after exposure to the UV light, respectively.

### 2.6. Viscosity Test of PUA and the Resin Formulations 

The viscosities of pure m-PUA and twelve resin formulations were measured using a DV3T rheometer (Brookfield Engineering AMETEK, Middleboro, MA, USA) equipped with spindle V-74 at room temperature. 

### 2.7. Hardness Measurement of PUA and the Resins

The surface hardness was measured using GS-792G Shore D type durometer (Teclock, Nagano Prefecture, Japan) according to ASTM D 2240, ISO 868. Cured PUA and twelve resins were tested after post-cure process. 

### 2.8. Compressive Test of PUA and the Resins

The compressive strength and modulus of the printed specimens at Φ5 × 10 mm were measured using an AGS-500G universal testing machine (SHIMADZU, Kyoto, Japan) with a load cell of 5000 N. According to ASTM D 965 and ISO 604, the crosshead speed was set to 1 mm min^−1^. Cured PUA and twelve resins were tested after post-cure process.

### 2.9. Contact Angle Measurement of PUA and the Resins

A contact angle goniometer (Tntec, Lunderskov, Denmark) was used to determine the surface contact angle. Then, a 5 μL water droplet was dropped on the surface of the post-cured specimens of the pure PUA and twelve resins. 

### 2.10. In Vitro Degradation Test of PUA and the Resins 

To evaluate the degradation rate of the printed resins, an accelerated hydrolytic degradation test was performed for shortening the experiment time. Specimens were dried at 60 °C for 24 h in advance. Specimens were prepared into blocks in the size of 10 × 10 × 2 mm^3^. Specimens were immersed in 15 mL 2 M NaOH at 60 °C for 2, 6, 8, 24 and 48 h. Five specimens were taken from each group at every time period and washed with distilled water. Then, the specimens were dried at 60 °C for 24 h. The remaining mass was calculated according to the following equation:

$$\mathrm{Remaining}\;\mathrm{mass}\;\left(\%\right)=100-\frac{\left(M_{i}-M_{f}\right)\times 100}{M_{i}}$$

where *M_i_* is the initial mass of the sample and *M_f_* is the final mass of the sample after degradation.

### 2.11. Scanning Electron Microscope (SEM) of the Printed Scaffolds

To reveal the surface morphology of the printed scaffolds, a JSM-7600F Schottky field emission scanning electron microscope (SEM; JEOL, Tokyo, Japan) was used. The scaffolds printed with PUA530-PEGDA-PPG (70:30:0), PUA800-PEGDA-PPG (70:30:0) and PUA1000-PEGDA-PPG (70:30:0) were observed.

### 2.12. Cytotoxicity Test of PUA and the Resins by MTT Assay 

To evaluate the in vitro cytotoxic effects of any leachable by-products from the polymerized PUA, the extract test was carried out using 3-(4,5-dimethylthiazol-2-yl)-2,5-diphenyltetrazolium bromide (MTT) assay according to ISO 10993-5. Briefly, mouse fibroblasts (L929) were cultured in minimum essential medium (MEM) (Gibco) containing 10% fetal bovine serum (SBF) and 1% penicillin streptomycin. Following ISO standards 10993-12 the extract was prepared by soaking samples in culture medium at a concentration of 3 cm^2^ mL^−1^. The extraction was carried out at 37 °C for 24 h. Cells were seeded with the concertation of 10^4^ cells 100 μL^−1^ in a 96-well plate. To ensure the cytotoxic response of the specimens provides an appropriate response of the cells, the negative control and the positive control were also analyzed. Cells cultured in 0.4% phenol in culture medium were the positive control which provides a reproducible cytotoxic response and cells in culture medium only were the negative control which provides a background response of the cells. After cells seeded for 24 h, all the culture mediums were removed and replaced with 100 μL extract. Finally, the culture medium was removed, and 50 μL MTT solution was added in each well. After incubation at 37 °C for 3 h, the MTT solution was aspirated and 100 μL isopropanol was added to each well to dissolve the purple formazan. After shaking for 30 min, absorbance was measured using Multiskan Spectrum ELISA reader (Thermo Fisher Scientific, Waltham, MA, USA) at 570 nm. The cell viability was calculated according to the following equation:

$$\mathrm{Viab}.\;\%=\frac{100\times (OD_{570t}-OD_{570b})}{OD_{570n}-OD_{570b}}$$

where *OD*_570*t*_ is the value of the measured optical density of the test well, *OD*_570*n*_ is the value of the measured optical density of the negative control, and *OD_570b_* is the mean value of the measured optical density of the blanks.

### 2.13. Statistical Analysis 

Statistical differences among the groups were analyzed using one-way analysis of variance (ANOVA). The differences of degree of conversion before and after post-curing process were analyzed using independent-samples T-test. *p*-values < 0.05 were considered statistically significant.

## 3. Results 

### 3.1. NMR and FTIR Spectrum of PCL Diol and PUA

**Figure 4:**
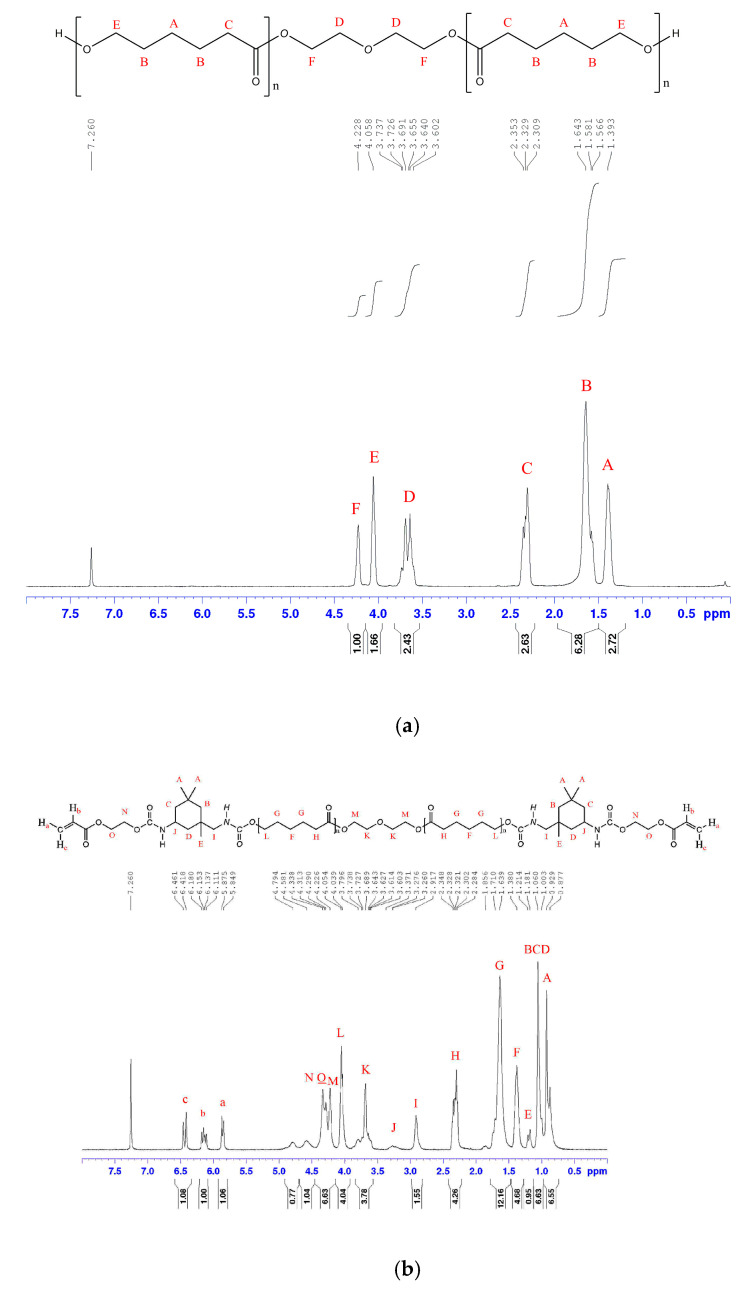
NMR spectra of PCL530 diol (**a**) and m-PUA530 (**b**).

^1^H-NMR spectrum was commonly used to ascertain the number average molecular weight (M_n_) of the PCL diols. The average number of CL units per PCL diol was calculated by comparing the peak integral of the methylene bridges of diethylene glycol (peak *D* at 3.6–3.8 ppm and peak *F* 4.2–4.3 ppm) with the peak integral of the methylene bridges of the PCL backbone (peak *A* at 1.3–1.5 ppm, peak *B* at 1.5–1.8 ppm, peak *C* at 2.2–2.4 ppm and peak *E* at 24.0–4.1 ppm) [13]. The results from ^1^H-NMR analysis of PCL diol were summarized in Table 2. In addition, the ^1^H-NMR spectra of PCL530 diol and m-PUA530 is shown in Figure 4. The peaks at 0.8–1.0 ppm (A), 1.0–1.1 ppm (B, C and D), 1.1–1.3 ppm (E), 2.8–3.0 ppm (I) and 3.2–3.3 ppm (J) correspond to IPDI [14]. The peak within 4.25–4.4 ppm (N and O) is the methylene bridges between the urethane groups and the acrylate. Three peaks at 5.8–5.9 ppm (A), 6.1–6.2 ppm (B) and 6.4–6.5 ppm (C) which correspond to vinyl groups in acrylate suggest the presence of acrylic groups [15]. 

**Figure 5:**
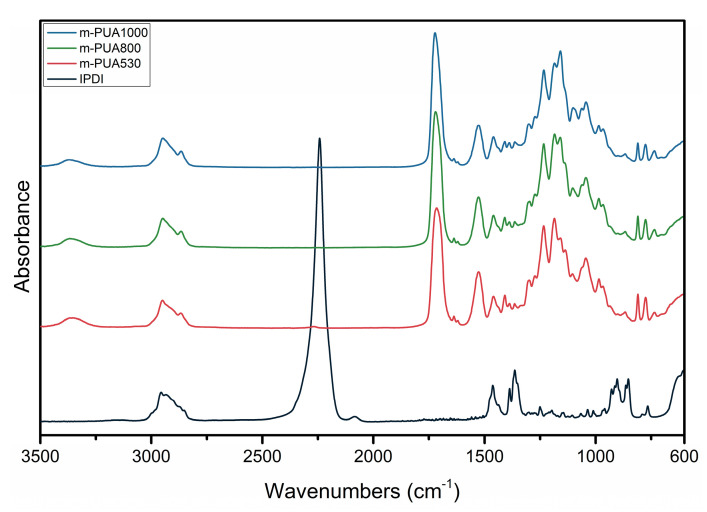
FTIR spectra of IPDI, m-PUA53, m-PUA800 and m-PUA1000.

Figure 5 shows the FTIR spectra of m-PUA and IPDI as a contrast. The FTIR spectra registered peaks of urethane group appear at 3360 cm^−1^ (NH, hydrogen-bonded), 2860–2950 cm^−1^ (CH_2_ and CH_3_), 1727 cm^−1^ (C=O) and 1526 cm^−1^ (N-monosubstituted amides), respectively. Peaks at 1639 cm^−1^ and 1617 cm^−1^ correspond to the acrylic group. The peak at 2250–2280 cm^−1^ attributes to NCO stretching vibration from IPDI [16]. The absence of the N=C=O peak indicates that there is a negligible amount of free N=C=O [10]. There was no peak of NCO stretching vibration for m-PUA530, m-PUA800 and m-PUA1000 after the synthesis. 

### 3.2. Viscosity of PUA and the Resin Formulations

**Figure 6:**
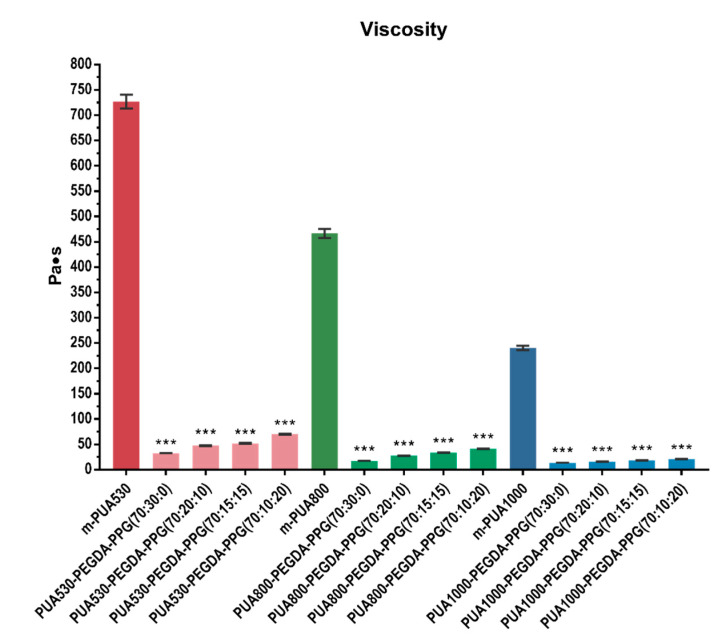
Viscosities of pure m-PUA and twelve unpolymerized resin (*** *p* < 0.001).

The results of the viscosity test were shown in Figure 6. Adding thirty percent of PEGDA and PPG was found to decrease the viscosities of the resins significantly (*p* < 0.001). PUA blended with 30% of PEGDA shows the lowest viscosity in each group.

### 3.3. Calculation of Degree of Conversion of the Printed Resins

Table 3 shows the results of the printed resins before and after the post-curing process. After post-curing, the degree of conversion of all the resins increased.

### 3.4. Hardness Measurement of PUA and the Resins

**Figure 7:**
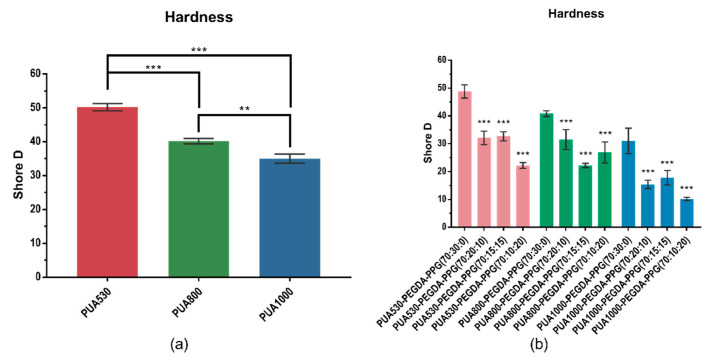
Hardness values of pure PUA (**a**) and twelve different resins (**b**) (** *p* < 0.01, *** *p* < 0.001).

Figure 7 shows hardness values of polymerized PUA and twelve different printed resins. PUA530 shows the highest value among all the materials. As molecular weights of PUA increased, the hardness value of specimens decreased. It was found that the addition of PPG significantly decreased (*p* < 0.001) the surface hardness. 

### 3.5. Compressive Test of PUA and the Resins

**Figure 8:**
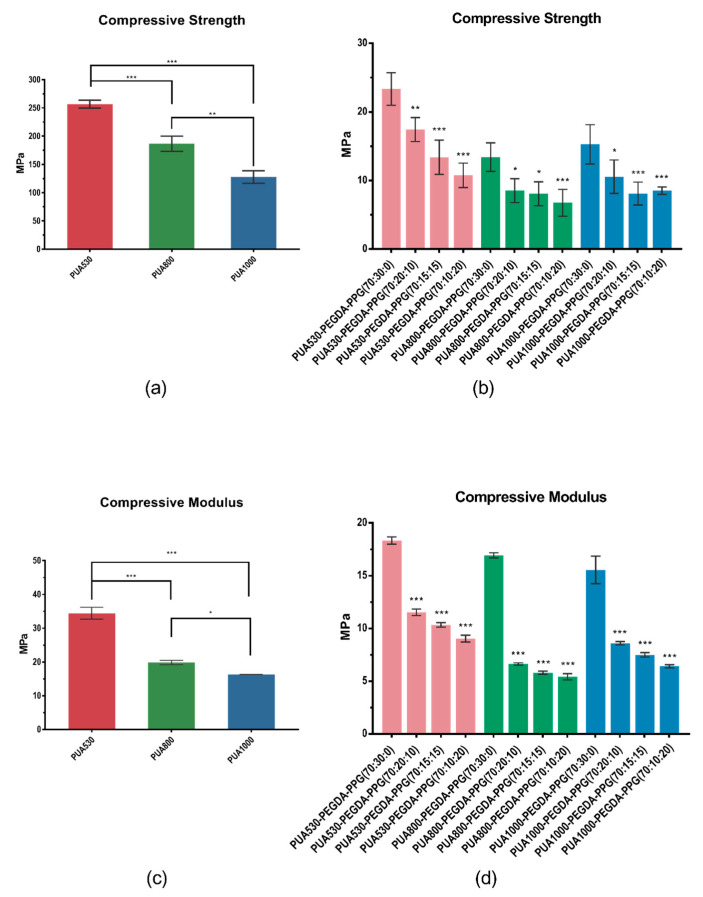
The results of the compressive tests (* *p* < 0.05, ** *p* < 0.01, *** *p* < 0.001). Compressive strength of pure PUA (**a**) and twelve resins (**b**). Compressive modulus of pure PUA (**c**) and twelve resins (**d**).

As shown in Figure 8a, the compressive strength of polymerized PUA530, PUA800 and PUA1000 were 256.52 MPa, 186.38 MPa and 127.66 MPa, respectively. As for compressive modulus, the compressive modulus of pure PUA530, PUA800 and PUA1000 were 34.43 MPa, 19.82 MPa and 16.29 MPa, respectively (Figure 8c). Compare to the formula PUA-PEGDA-PPG (70:30:0), the addition of PPG significantly decreased (*p* < 0.01) the compressive strength and modulus. Generally, as the percentage of PPG increased, the compressive strength and modulus decreased. 

### 3.6. Contact Angle Measurement of PUA and the Resins

**Figure 9:**
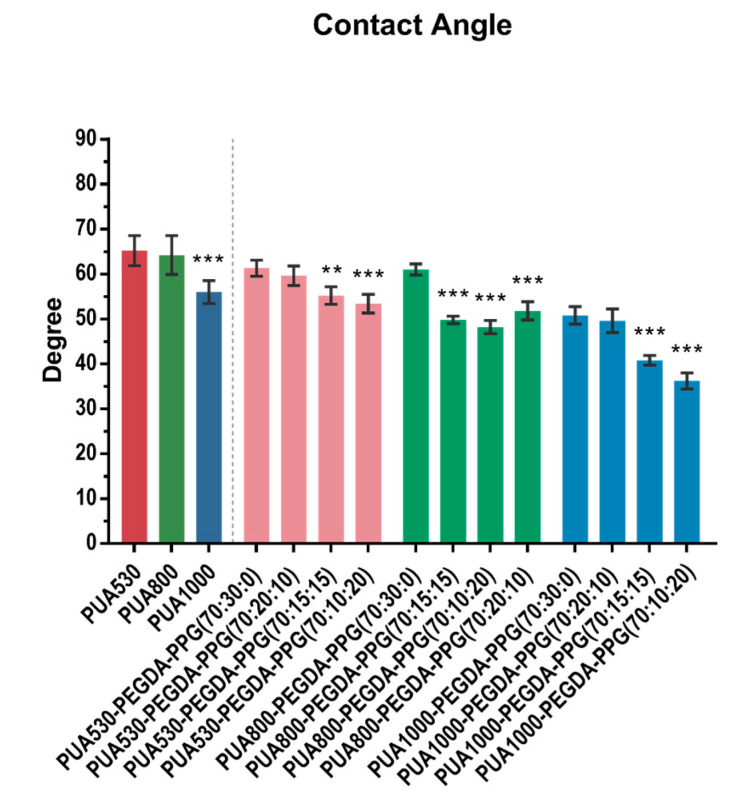
Contact angle of pure PUA and twelve different resins (** *p* < 0.01, *** *p* < 0.001).

The contact angle measurements of three polymerized PUA and twelve printed resins are shown in Figure 9. PUA1000 showed the significantly lowest (*p* < 0.001) degree among three pure PUAs. After adding 30% PEGDA and PPG, the contact angle lowered, and the resins containing higher percent of PPG showed lower degree. For faster degradation rate of the materials and better cell attachment, lower contact angle is generally prospected.

### 3.7. Degradation Test of PUA and the Resins In Vitro

**Figure 10:**
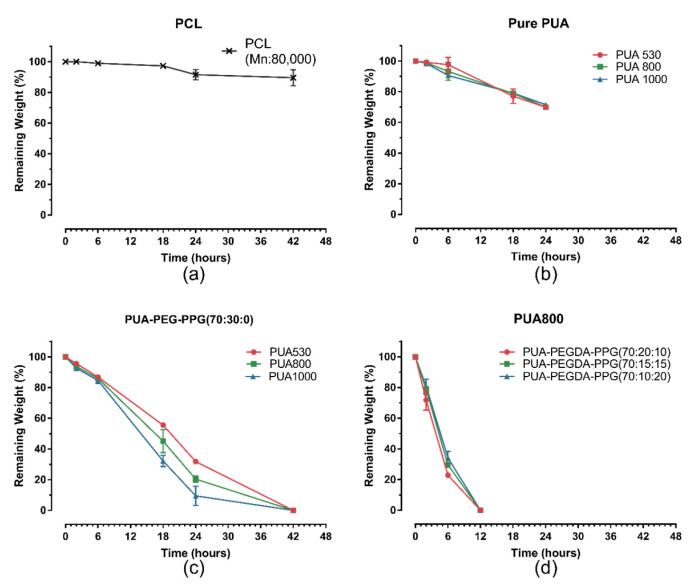
The remaining weights of commercialized PCL (**a**), pure PUA (**b**), formula PUA-PEGDA-PPG (70:30:0) (**c**) and PUA800 (**d**) formulated in three different formula evaluated at different time.

The remaining weights of commercialized PCL, pure PUA, PUA530-PEGDA-PPG (70:30:0), PUA800-PEGDA-PPG (70:30:0), PUA800-PEGDA-PPG (70:20:10), PUA800-PEGDA-PPG (70:15:15), PUA800-PEGDA-PPG (70:10:20) and PUA1000-PEGDA-PPG (70:30:0) evaluated at different time were shown in Figure 10. The degradation rate of commercialized PCL was the slowest and three PUA were the second one. The weights of pure PUA lost about 30% after 24 h. The resin without PPG degraded completely in 42 h, while, the three formulas containing PPG sharply lost the weight during the period of 2–6 h and completely degraded within 12 h.

### 3.8. Scanning Electron Microscope (SEM) of the Scaffolds 

**Figure 11:**
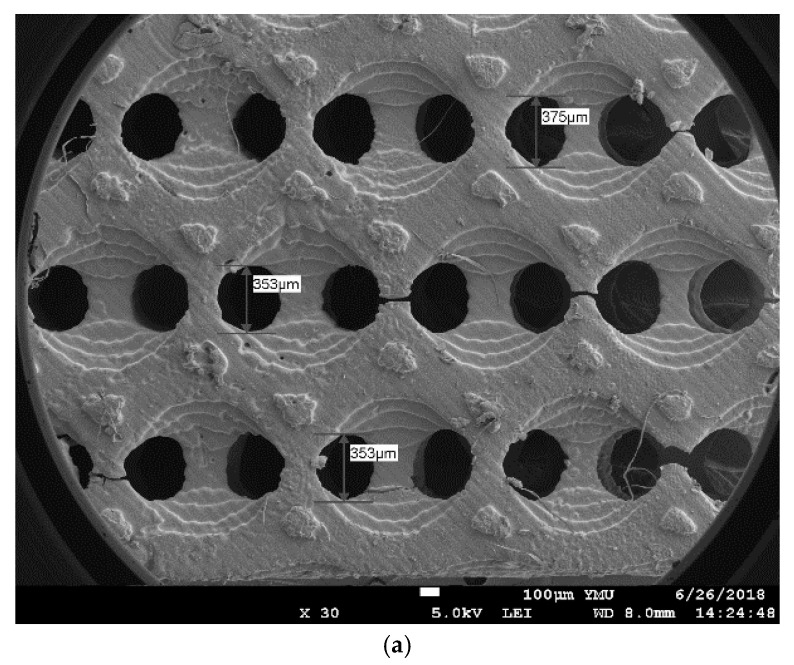

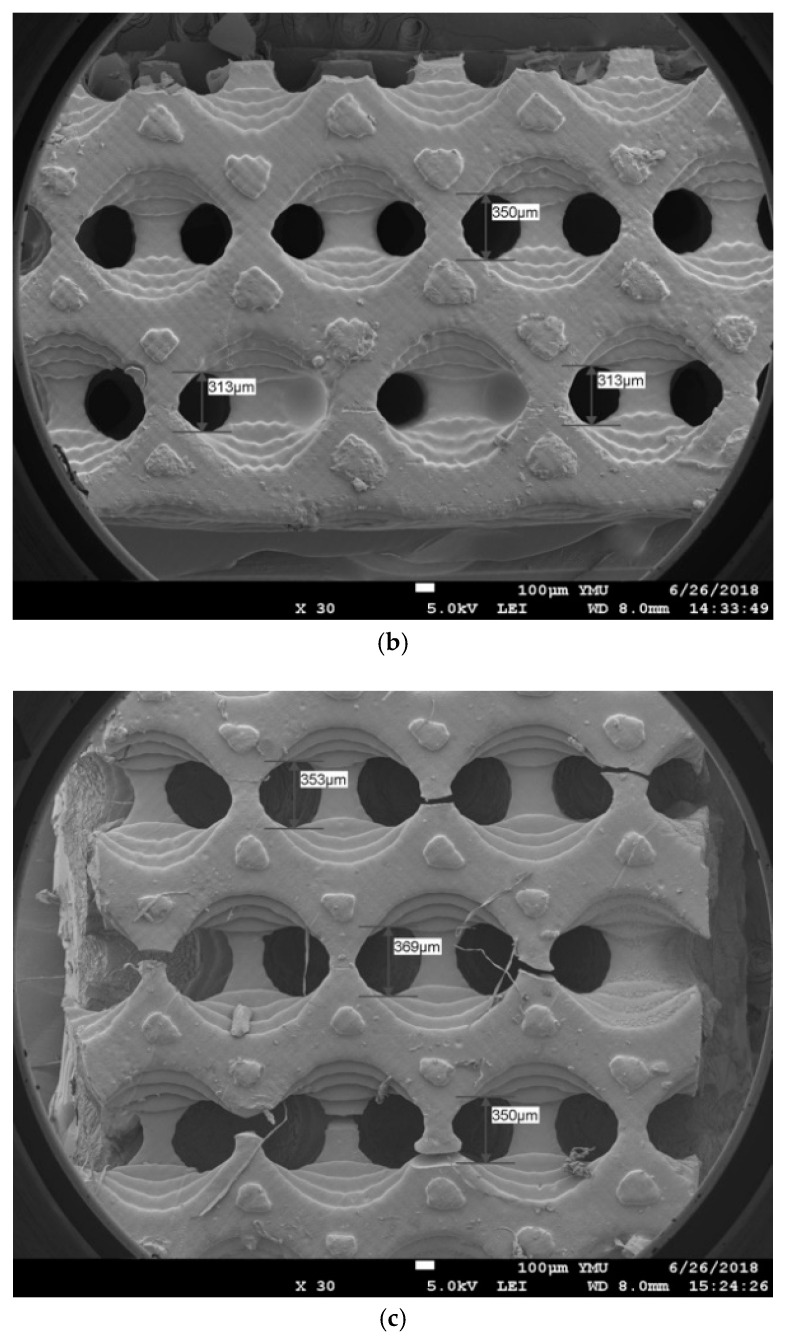
SEM images of printed scaffolds. (**a**) is the top surface of PUA530-PEGDA-PPG (70:30:0), (**b**) is the top surface of PUA800-PEGDA-PPG (70:30:0), (**c**) is the top surface of PUA1000-PEGDA-PPG (70:30:0).

The SEM images of printed scaffolds of PUA530-PEGDA-PPG (70:30:0), PUA800-PEGDA-PPG (70:30:0) and PUA1000-PEGDA-PPG (70:30:0) were presented in Figure 11. Figure 11a–c show the top surface of x-y plane of printed scaffolds for PUA530-PEGDA-PPG (70:30:0), PUA800-PEGDA-PPG (70:30:0) and PUA1000-PEGDA-PPG (70:30:0), respectively. The sizes of the connections were various from 313 to 375 μm. 

### 3.9. Cytotoxicity Test by MTT Assay

**Figure 12:**
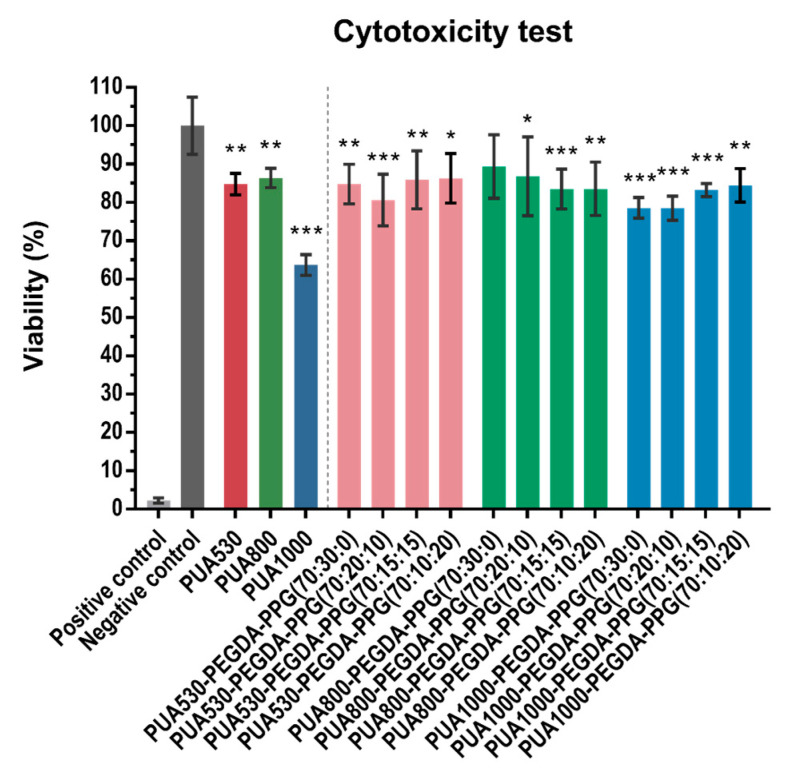
Cell viability of L929 after incubation in different extractions of pure PUA and twelve resins, based on the result of MTT assay (* *p* < 0.05, ** *p* < 0.01, *** *p* < 0.001).

The results of the cytotoxicity test with MTT assay were shown in Figure 12. The results of PUA530, PUA800 and PUA1000 were 84.82%, 86.42% and 63.71%, respectively, all of which were significantly lower (*p* < 0.05) than negative control. Comparing to the negative control, there were significant differences (*p* < 0.05), except for PUA800-PEGDA-PPG (70:30:0).

## 4. Discussion

The chemical structures of synthesized PUA were characterized with NMR and FTIR spectrometer. The average number of CL units per PCL diol of PCL800 diol and PCL1000 diol was fewer than as designed, so the M_n_ is lower than we expected. The intramolecular transesterification occurring during the ring-opening polymerization of ε-CL at high temperature might cause this result [17]. 

Generally speaking, the higher the molecular weight is, the higher the viscosity would be. In contrast, m-PUA1000, which with higher molecular weight, showed lower viscosity than m-PUA530. The viscosity of a monomer results in the relative density of the hydrogen bonds and intermolecular chain entanglements [18]. In per unit of PUA, the m-PUA1000 has relatively fewer intermolecular and intramolecular hydrogen bonds created from urethane groups (-OCONH-) than m-PUA530, so the viscosity of m-PUA1000 is lower than m-PUA530. According to our preliminary test, at least 30% substitution of PUA could effectively decrease the viscosity and allowed it to be printed, so 30% of PUA was substituted with PEGDA and PPG in this study. Our results show that the viscosities decreased by 10 times after adding 30% of PEGDA. Although several papers have mentioned that the viscosities of resins would affect the results of printed model [19], there is no study indicated that what range of the viscosity of resins exactly is most ideal. According to the commercial products, the viscosities commonly within the rage of 0.4–3.5 Pa·s. All the resins were printed and could form into simple shapes, such as cylinders or cubes although there is no viscosity being comparable to the viscosity of commercialized products. While for the complex structures, resins with lower viscosity shows better performance. Thus, PUA530-PEGDA-PPG (70:30:0), PUA800-PEGDA-PPG (70:30:0) and PUA1000-PEGDA-PPG (70:30:0) were further printed into scaffold.

The degree of conversion (DC) is a crucial factor in photo-cured polymers. It directly affects the mechanical properties of the composites. The degree of conversion is determined by the percentage of the remaining concentration of the C=C double bonds in the acrylic group in a polymerized sample relative to the total number of C=C bonds in the acrylic group in the uncured materials. With post-curing processes, the conversion of the photopolymer could be raised. The DC of printed samples before the post-curing process is around 65–70%. After the post-curing process, all the DC are over 80%. 

Mechanical properties were evaluated by carrying out the compressive test and the hardness measurement. The results of the compressive test reveal that the higher the molecular weight is, the lower the mechanical properties would be. The soft segments are composed of diol part, hence the raising of the molecular weight of diols would lead to the losing of rigidity and strength [20]. It is clear that mechanical properties of the resins are decreased by adding PPG in comparison with the pure PUA, because the mechanical properties for photo-curable materials would be improved with higher DC [21]. PPG was added as a plasticizer. There is no acrylic group tipping on the end of PPG, so PPG could not participate in the polymerization. The results indicate that the addition of PPG could decrease the compressive modulus, as we expected. The hardness of twelve resins are from 10.2 to 48.8 which similar to the hardness of cartilage (Shore D 30) [19]. As for the compressive test, the compressive strength of twelve resins are from 6.8 to 23.4 MPa which within the range of cartilage (2.9–40 MPa); the compressive modulus of twelve resins are from 5.4 to 18.3 MPa which close to the range of cartilage (4.5–24 MPa) [22]. Therefore, we believe that these resins could be applied for cartilage scaffolds in the future.

In order to assess hydrolysis rate of the printed resins, the degradation test was performed. It is time-consuming to carry out the degradation test using the real time-scale, as a consequence, an accelerated hydrolytic degradation test was performed to speed up the process in this study [7]. However, there is no specific equation for converting the results of the accelerated degradation to that of real time in vivo. A commercialized PCL (Mn: 80,000) was used as a comparison. The degradation rate of PU is mainly determined by the ester groups in the diol parts, because hydrolysis of the soft segments is much faster than the hard segments. The result obtained by Chern et al. [23] indicated that the weight loss of PCL in the Mn: 80,000 was around 4.77% after 15 weeks. Among the weight loss of three kinds of pure PUA, the result of PUA1000 loses the most weight. In theory, the degradation rate of PCL decreased as the length of the chain increases. However, the trend of the weight loss in this study is: PUA1000 > PUA800 > PUA530. This result might be caused by the hydrophilicity and crosslink density. Because the mechanism of hydrolytic degradation of polyurethanes is the hydrolysis of the ester and urethane groups, the water uptake becomes one of key factors. The degree of water absorption can be characterized by the hydrophilicity of polyurethanes as well. 

To ensure the extracts of the pure PUA and twelve resins are non-toxic, cytotoxicity test was carried out. According to ISO10993-5, if the cell viability was lower than 70%, the material has a cytotoxic potential. The viabilities of all the materials are over 70%, except for pure PUA1000. The sources of cytotoxic damage might be the results of residual organic solvent from the synthesis, the leaching of unreacted monomer, the degradation products and photoinitiator [22]. TPO will convert into free radicals that participate in the photopolymerization, after exposure to light. Thus, after printing and post-curing, the remaining TPO would be negligible.

The porous structures of printed scaffolds were observed using SEM. The scaffolds were printed using only PUA-PEGDA-PPG (70:30:0), because the viscosities of these resin formula is the lowest among other resin formulas. Resins with lower viscosity was expected to have better performance in the printed scaffolds because they are easier to flow in and out of the printed model during the peeling actions. Although the viscosity of PUA530-PEGDA-PPG (70:30:0) (32.24 Pa·s) is much higher than PUA800-PEGDA-PPG (70:30:0) (17.07 Pa·s) and PUA1000-PEGDA-PPG (70:30:0) (13.85 Pa·s), it could still be printed well; the higher mechanical properties could resist the peeling force during the layer by layer printing. However, the cracks on the scaffolds might be caused by the cleaning process. The scaffold of PUA1000-PEGDA-PPG (70:30:0) was so fragile that there were more cracks after the cleaning process. 

## 5. Conclusions

The PCL-based polyurethane acrylate prepolymers were successfully synthesized with PCL diol, IPDI and 2-HEA and formulated into twelve resin mixtures. The addition of different ratios of PEGDA and PPG can tune the mechanical properties of the resin mixtures that are similar to the cartilage. The twelve printed resins showed advantages, such as cytocompatibility and degradability. PUA530-PEG-PPG (70:30:0), PUA800-PEG-PPG (70:30:0), and PUA1000-PEG-PPG (70:30:0) were successfully printed into customized tissue scaffolds with precise pore size. In the future, these resins may have great potential for customized tissue engineering.

## Figures and Tables

**Table 1:** The different percentages of polyurethane acrylate (PUA), poly (ethylene glycol) diacrylate (PEGDA), and propylene glycol (PPG) for printable formula of the resin.

Name	Monomer		
	**PUA530**	**PEG600DA**	**PPG**
**PUA530-PEG-PPG (70:30:0)**	70%	30%	0%
**PUA530-PEG-PPG (70:20:10)**	70%	20%	10%
**PUA530-PEG-PPG (70:15:15)**	70%	15%	15%
**PUA530-PEG-PPG (70:10:20)**	70%	10%	20%
	**PUA800**	**PEG600DA**	**PPG**
**PUA800-PEG-PPG (70:30:0)**	70%	30%	0%
**PUA800-PEG-PPG (70:20:10)**	70%	20%	10%
**PUA800-PEG-PPG (70:15:15)**	70%	15%	15%
**PUA800-PEG-PPG (70:10:20)**	70%	10%	20%
	**PUA1000**	**PEG600DA**	**PPG**
**PUA1000-PEG-PPG (70:30:0)**	70%	30%	0%
**PUA1000-PEG-PPG (70:20:10)**	70%	20%	10%
**PUA1000-PEG-PPG (70:15:15)**	70%	15%	15%
**PUA1000-PEG-PPG (70:10:20)**	70%	10%	20%

**Table 2:** The number average molecular weights (M_n_) and composition of PCL530, PCL800 and PCL1000 from ^1^H-NMR.

Monomers	Targeted M_n_ (Da)	^1^H-NMR M_n_ (Da)	The Number of CL Units per DEG Molecule
PCL530 diol	530	548.32	3.88
PCL800 diol	800	681.63	5.05
PCL1000 diol	1000	793.42	6.03

**Table 3:** Degree of conversion of resins before and after the post-curing process at 60°.

Resins	DC (Before Post-Curing)	DC% (After Post-Curing)
PUA530-PEGDA-PPG (70:30:0)	65.55 ± 1.18 ^d,e,f^	84.28 ± 0.65 ^e,f,g^
PUA530-PEGDA-PPG (70:20:10)	68.60 ± 1.18 ^b,c,d,e,f^	87.09 ± 0.09 ^c,d,e,f,g^
PUA530-PEGDA-PPG (70:15:15)	68.39 ± 0.97 ^b,c,d,e,f^	83.42 ± 0.58 ^e,f,g^
PUA530-PEGDA-PPG (70:10:20)	68.74 ± 1.04 ^b,c,d,e,f^	83.09 ± 3.78 ^e,f,g^
PUA800-PEGDA-PPG (70:30:0)	68.34 ± 0.62 ^b,c,d,e,f^	90.11 ± 0.80 ^b,c,d,e^
PUA800-PEGDA-PPG (70:20:10)	70.93 ± 2.23 ^b,c,d,e,f^	92.16 ± 1.57 ^b,c,d^
PUA800-PEGDA-PPG (70:15:15)	64.54 ± 2.64 ^d,e,f^	91.48 ± 1.07 ^b,c,d,e^
PUA800-PEGDA-PPG (70:10:20)	68.26 ± 1.31 ^b,c,d,e,f^	91.83 ± 4.07 ^b,c,d^
PUA1000-PEGDA-PPG (70:30:0)	72.27 ± 0.99 ^b,c,d^	88.92 ± 0.58 ^b,c,d,e,f^
PUA1000-PEGDA-PPG (70:20:10)	65.30 ± 1.31 ^d,e,f^	89.42 ± 1.48 ^b,c,d,e^
PUA1000-PEGDA-PPG (70:15:15)	71.43 ± 1.39 ^b,c,d,e^	90.90 ± 0.07 ^b,c,d,e^
PUA1000-PEGDA-PPG (70:10:20)	66.64 ± 0.58 ^c,d,e,f^	84.72 ± 0.50 ^d,e,f,g^

The same superscript small letters indicate a homogeneous subset (columns) (*p* < 0.05).
